# A Mouse Model of Glycogen Storage Disease Type IX-Beta: A Role for *Phkb* in Glycogenolysis

**DOI:** 10.3390/ijms23179944

**Published:** 2022-09-01

**Authors:** Charles J. Arends, Lane H. Wilson, Ana Estrella, Oh Sung Kwon, David A. Weinstein, Young Mok Lee

**Affiliations:** 1 Department of Pediatrics, University of Connecticut School of Medicine, Farmington, CT 06030, USA; 2 Department of Genetics and Genome Sciences, University of Connecticut Health Center, Farmington, CT 06030, USA; 3 Gene Therapy Program, Department of Medicine, Perelman School of Medicine, University of Pennsylvania, Philadelphia, PA 19104, USA; 4 Department of Kinesiology, University of Connecticut, Storrs, CT 06269, USA; 5 Department of Orthopaedic Surgery and Center on Aging, University of Connecticut Health Center, Farmington, CT 06030, USA

**Keywords:** glycogenolysis, hepatomegaly, hypoglycemia, ketosis, glucose

## Abstract

Glycogen storage disease type IX (GSD-IX) constitutes nearly a quarter of all GSDs. This ketotic form of GSD is caused by mutations in phosphorylase kinase (PhK), which is composed of four subunits (α, β, γ, δ). PhK is required for the activation of the liver isoform of glycogen phosphorylase (PYGL), which generates free glucose-1-phosphate monomers to be used as energy via cleavage of the α -(1,4) glycosidic linkages in glycogen chains. Mutations in any of the PhK subunits can negatively affect the regulatory and catalytic activity of PhK during glycogenolysis. To understand the pathogenesis of GSD-IX-beta, we characterized a newly created PHKB knockout (*Phkb*^−/−^) mouse model. In this study, we assessed fasting blood glucose and ketone levels, serum metabolite concentrations, glycogen phosphorylase activity, and gene expression of gluconeogenic genes and fibrotic genes. *Phkb*^−/−^ mice displayed hepatomegaly with lower fasting blood glucose concentrations. *Phkb^−/−^* mice showed partial liver glycogen phosphorylase activity and increased sensitivity to pyruvate, indicative of partial glycogenolytic activity and upregulation of gluconeogenesis. Additionally, gene expression analysis demonstrated increased lipid metabolism in *Phkb^−/−^* mice. Gene expression analysis and liver histology in the livers of old *Phkb^−/−^* mice (>40 weeks) showed minimal profibrogenic features when analyzed with age-matched wild-type (WT) mice. Collectively, the *Phkb^−/−^* mouse recapitulates mild clinical features in patients with GSD-IX-beta. Metabolic and molecular analysis confirmed that *Phkb^−/−^* mice were capable of sustaining energy homeostasis during prolonged fasting by using partial glycogenolysis, increased gluconeogenesis, and potentially fatty acid oxidation in the liver.

## 1. Introduction

Glycogen storage disease type IX (GSD-IX, OMIM306000) constitutes the largest subgroup of glycogen storage diseases [1]. It is caused by a deficiency in phosphorylase kinase (PhK) enzyme resulting in the insufficient break down of glycogen [1,2]. PhK plays an integral part in glucose metabolism in the liver by phosphorylating the liver glycogen phosphorylase (PYGL) that switches from phosphorylase b to phosphorylase a by a reversable phosphorylation of serine residue 15 [3,4]. PhK has been known to be associated with the regulatory mechanism, the direct transfer of phosphate from adenosine triphosphate (ATP) to the substrate serine, triggering a conformational switch from the inactive (phosphorylase b) form to the active (phosphorylase a) form, catalyzing the breakdown of glycogen into glucose 1-phosphate (G1P) monomers [5]. During an interprandial state, PhK stimulated by glucagon promotes glycogenolysis to produce glucose from glycogen through glycogen phosphorylase and maintains normal blood glucose levels. The impaired glycogen breakdown due to the defects in PYGL or PhK causes glycogen accumulation, leading to hepatomegaly, ketotic hypoglycemia, and growth retardation in affected patients with GSD-IX [1,6,7,8,9].

Although GSD-IX is considered a mild form of GSD, severe liver complications including liver failure, fibrosis and cirrhosis can occur [1,6,10,11,12,13]. During infancy, combined with clinical and laboratory evaluation, biochemical test of liver enzymes (ALT and AST) and histological analysis by liver biopsy can identify liver damage and diagnose GSD-IX [14]. Different types of GSD require different treatments. Due to similarity of phenotypes such as hypoglycemia, hepatomegaly, and elevated liver enzymes, Sanger DNA sequencing or next generation sequencing(NGS) including exome sequencing have been widely used to find precise mutations to design targeted therapies for specific types of GSD [14].

PhK is 1.3 MDa hexadecameric holoenzyme and consists of four isoforms of the α-, β-, or γ- subunits. Only the γ subunit is known to possess catalytic activity, while the others serve regulatory functions [15]. There is a wide spectrum of disease presentations in GSD-IX patients and each subunit is linked to different subtype of GSD-IX including, α1; PHKA1(OMIM #311870), GSD-IXd (muscle), α2; PHKA2 (OMIM #300798), GSD-IXa (liver), β; PHKB (OMIM #172490), GSD-IXb (liver and muscle), and γ2; PHKG2(OMIM #172471), GSD-IXc (liver and muscle) [1,8,16,17].

While GSD IX is likely the most common form of GSD, basic science research with disease models has focused on the comparably more severe types of GSD including GSD I [18,19] and GSD-III [20]. For GSD-IX, a mouse model for GSD IX gamma 2 has recently been characterized [21]. The Phkg2 knockout mouse model recapitulated the liver specific phenotype of GSD-IXγ patients, showing significantly reduced phosphorylase kinase activity with elevated glycogen accumulation and early signs of liver damage at 3 months of age. As serious complications such as liver fibrosis and cirrhosis had been reported in GSD-IXc [22], longer-term monitoring on this model will be required for better understanding of this type of GSD-IX [21]. In contrast to the GSD-IXc, patients with PHKB deficiency typically show milder symptoms [1,13,23,24], and this has led to less attention being devoted to investigating the pathological processes in GSD-IXb. Thus far, except for hepatomegaly, other details of the disease mechanism, including long-term effects of glycogen accumulation in the liver of patients with GSD-IXb, have not been looked at leaving the mechanisms leading to the less severe phenotype still unknown.

*PHKB* is a large gene spanning just over 239 kb and containing 33 exons [6]. Mutations in the *PHKB* gene are autosomal recessive and lead to deficiencies in the β subunit, affecting both liver and muscle. Several mutations have been found in the *PHKB* gene in GSD-IX patients which lead to enzyme deficiency in the liver and muscle including independent nonsense mutations, a single-base insertion, splice-site mutations and an extensive intragenic deletion [24]. Previously, we reported on the deficiency of the liver glycogen phosphorylase enzyme in a murine model [25]. However, the relationship between enzyme deficiency, glycogen and glucose metabolism, and the potential long-term complications in the pathogenesis of ketotic GSDs is not well understood. Therefore, the purpose of this study is to ascertain the distinct effect this enzymatic defect of glycogenolysis has on glycogen metabolism using a *Phkb* deficient mouse model. In this study, we characterize the newly created *PHKB* knockout mouse, a representation of GSD-IX-beta (MIM#261750). We found GSD-IX-beta mice exhibited a mild ketotic phenotype, with minimal profibrogenic features, and demonstrate limited glycogenolytic ability to utilize excessive glycogen stores during prolonged fasting.

## 2. Results

### 2.1. Characterization of Phosphorylase Kinase Beta Deficiency: A Model for GSD-IX-Beta

**Figure 1:**
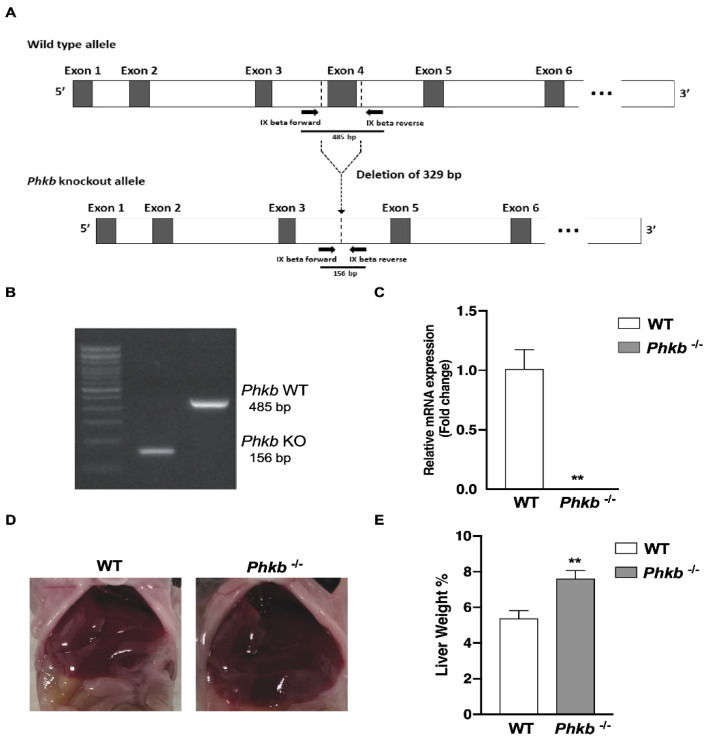
Generation of Phosphorylase kinase beta deficient mouse. (**A**) Schematic representation of the Phkb-knockout and WT alleles. Deletion of 329 bp of exon 4 and flanking sequence of the phosphorylase kinase beta (*Phkb*) gene generates *Phkb*-knockout allele without exon 4. Arrows indicate the primer set used for genotyping. (**B**) PCR amplification of two PCR fragments, 485 bp and 156 bp in length amplified genomic DNA of *Phkb^−/−^* and WT (*Phkb^+/+^*). (**C**) Relative mRNA expression (Fold change) of phosphorylase kinase beta subunit gene (*Phkb*) in wild-type (n = 5; WT, white bar) and *Phkb^−/−^* (n = 11, grey bar) mice. (**D**) Representative images of livers (WT, left; *Phkb*^−/−^ right). (**E**) Mean Liver weight to Body weight (LW/BW) percentage in young nonfasted (WT = 38, *Phkb*^−/−^ = 22) mice. mRNA expression and LW/BW percentage data were expressed as Mean ± SEM with ** *p* < 0.01.

PCR analysis confirmed the expected deletion of 329 bp of exon 4 of the *Phkb* gene (Figure 1A,B). Generation of *Phkb^−/−^* knockout mice exhibited no clear variation in physical growth or appearance compared to WT or heterozygous mice (data not shown). Relative mRNA expression of the *Phkb* gene was later confirmed by real-time qPCR (Figure 1C) in *Phkb*^−/−^ mice. We observed *Phkb*^−/−^ mice displayed hepatomegaly (Figure 1D) with elevated liver weight/body weight (LW/BW) percentages (7.57%) compared to WT (5.38%) mice (Figure 1E). No significant differences were observed in body weight or appearance between *Phkb*^−/−^ and WT mice (data not shown). To assess whether *Phkb* gene deficiency was associated with liver and metabolic characteristics associated with GSD-IX, we analyzed mouse fasting and liver phenotypes.

**Figure 2:**
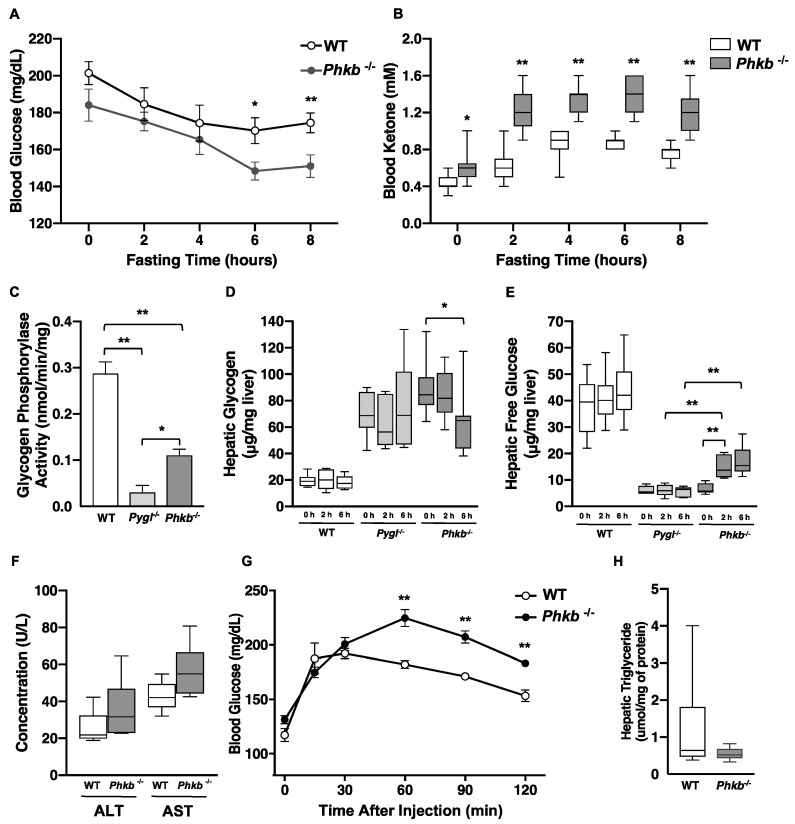
Phenotypical analysis of *Phkb^−/−^* mice. (**A**) Fasting glucose and (**B**) ketone tests were performed in (WT, n = 11, *Phkb^−/−^*, n = 9) mice. Blood glucose and ketone (Beta-hydroxybutyrate) levels were measured at 0, 2, 4, 6, and 8 h intervals; Mean blood glucose (mg/dL) in *Phkb^−/−^* mice (black dots) showed reduced baseline (0 h) blood glucose levels and significantly lower blood glucose levels at 6 (* *p* < 0.05) and 8 (** *p* < 0.01) hour intervals. WT (white dot) maintained blood glucose levels >160 mg/dL through fasting; fasting in *Phkb^−/−^* mice revealed significantly elevated ketone bodies (mM) at baseline (0 h, * *p* < 0.05) and at all fasting intervals (0 h; * *p* < 0.05, 2 h, 4 h, 6 h, and 8 h; ** *p* < 0.01 compared to WT mice. (**C**) Enzymatic activity of glycogen phosphorylase (PYGL) in wild-type (n = 6; WT, white bar), *Pygl^−/−^* (n = 6, light grey bar) and *Phkb^−/−^* (n = 6, grey bar) mice. (**D**) Hepatic glycogen and (**E**) free glucose levels at 0 h, 2 h, and 6 h of fasted WT (0 h, n = 11, 2 h, n = 13, and 6 h, n = 13), *Pygl^−/−^* (0 h, n = 7, 2 h, n = 5, and 6 h, n = 5), and *Phkb^−/−^* (0 h, n = 9, 2 h, n = 6, and 6 h, n = 7) mice. (**F**) Serum levels of ALT, AST in WT (n = 7) and *Phkb^−/−^* (n = 7) mice. (**G**) Pyruvate tolerance test for WT and *Phkb^−/−^* mice with mean blood glucose levels in mice (WT, n = 9 and *Phkb^−/−^* n = 6) through time intervals following pyruvate bolus injection (2.0 g/kg) after 16 h of fasting (0 h). (**H**) Hepatic triglyceride contents in WT (n = 9) and *Phkb^−/−^* (n = 10) mice. Fasting blood ketone, hepatic glycogen, hepatic free glucose, ALT, AST, and TG data shown as box and whisker plot, Min to Max. Fasting glucose levels and pyruvate tolerance test are expressed as the mean ± SEM. * *p* < 0.05 and ** *p* < 0.01.

Collection and analyses of blood glucose through 8 h of fasting (0, 2, 4, 6, 8) in 4–6 weeks old WT or *Phkb^−/−^* mice (WT = 11, *Phkb*^−/−^ = 9) showed that *Phkb*^−/−^ mice maintained reduced blood glucose levels compared to WT mice during fasting with significant differences at 6 and 8 h; severe hypoglycemia, however, did not develop (Figure 2A). Elevated concentrations of the ketone body, β-hydroxybutyrate (βOHB), were observed at baseline (t = 0) and at all fasting intervals (2–8 h) compared to WT mice (Figure 2B).

### 2.2. Phkb^−/−^ Mice Showed Partial Glycogenolysis and Endogenous Glucose Production

The rate limiting step in glycogenolysis is the conformational change of liver glycogen phosphorylase (PYGL) into the active form (phosphorylase a) capable of generate G1P by degradation of glycogen is triggered by phosphorylase kinase. Therefore, the level of glycogen phosphorylase activity and amount of glycogen accumulation can explain both phosphorylase kinase activity and its true contribution in glycogenolysis. To evaluate this, we compared hepatic enzymatic activity of glycogen phosphorylase (PYGL) in WT (n = 6), *Pygl*^−/−^ (n = 6) and *Phkb^−/−^* (n = 6) mice. Both *Pygl^−/−^* and *Phkb^−/−^* showed significantly reduced glycogen phosphorylase activity compared to WT (** *p* < 0.01); however, *Phkb^−/−^* mice still had 38% of WT mice’s activity, which is 3.7 times higher than *Pygl^−/−^* (* *p* < 0.05) (Figure 2C). It has been known that GSD-IX-beta affects muscle; thus, we also measured glycogen phosphorylase activity of muscle in WT (n = 4), *Pygl*^−/−^ (n = 3) and *Phkb^−/−^* (n = 4) mice. While *Phkb^−/−^* showed significantly reduced glycogen phosphorylase activity to WT and *Pygl*^−/−^, there is no significant difference between WT and *Pygl*^−/−^ (Figure S3). Hepatic glycogen content and free glucose production were monitored through 6 h of fasting in WT (0 h, n = 11, 2 h, n = 13, and 6 h, n = 13), *Pygl^−/−^* (0 h, n = 7, 2 h, n = 5, and 6 h, n = 5), and *Phkb*^−/−^ (0 h, n = 9, 2 h, n = 6, and 6 h, n = 7) mice (Figure 2D,E). Hepatic glycogen content in non-fasted and 2 h-fasted *Pygl^−/−^* and *Phkb^−/−^* mice showed significantly higher accumulation of glycogen (ug/mg) than WT mice (** *p*< 0.01). However, at 6 h, fasted *Phkb^−/−^* livers revealed decreased hepatic glycogen (* *p* < 0.05), which corresponded to an inversely related increase in hepatic glucose levels (** *p*< 0.01, Figure 2E) while WT livers experienced stabilization in glycogen content after the start of fasting with no significant changes of free glucose levels (>40 µg/mg) (Figure 2E). Due to significantly increased glycogen accumulation observed in *Phkb^−/−^* mice, we assessed ALT and AST levels. In contrast to *Pygl^−/−^* mice, which showed increased ALT and AST, *Phkb^−/−^* mice (n = 7) showed minimal increases without significance compared to WT mice (n = 7) (Figure 2F). These findings show *Phkb^−/−^* mice are capable of glycogen degradation for glucose production and explain the mild phenotype associated with this disease and the *Phkb^−/−^* model.

### 2.3. Alternatively Upregulated Pathways: Gluconeogenesis and Fatty Acid Metabolism

**Figure 3:**
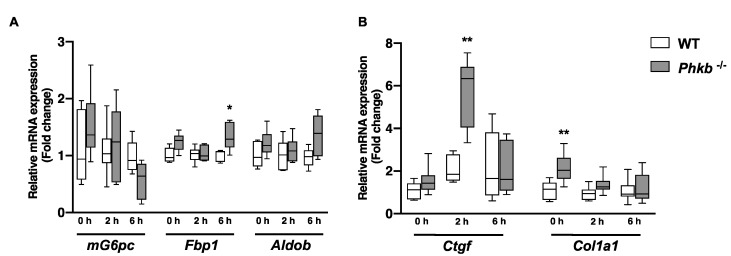
mRNA expression profile of gluconeogenic genes and fibrotic genes in WT and *Phkb^−/−^* mice. Quantification of hepatic mRNA for (**A**) gluconeogenic genes (*mG6Pc, Fbp1*, and *Aldob*), WT (0 h, n = 5, 2 h, n = 7, and 6 h, n = 5) and *Phkb^−/−^* (0 h, n = 11, 2 h, n = 6, and 6 h, n = 5) mice. (**B**) fibrosis related genes (*Ctgf* and *Col1a1*), WT (0 h, n = 5, 2 h, n = 7, and 6 h, n = 7) and *Phkb*^−/−^ (0 h, n = 11, 2 h, n = 6, and 6 h, n = 7) mice. Data represent the mean ± SD. * *p* < 0.05 and ** *p* < 0.01. Abbreviations: *mG6Pc*, mouse Glucose-6-phosphatase-*α*, *Fbp1*, Fructose-bisphosphatase 1, *Aldob*, Aldolase b. *Ctgf*, Connective tissue growth factor. *Col1a1*, Type I collagen.

As *Phkb^−/−^* mice showed limited breakdown of glycogen to produce glucose, we further investigated alternative pathways for energy production. While glycogenolysis involves the formation of glucose molecules from the degradation of glycogen, gluconeogenesis forms glucose from non-carbohydrate substrates such as pyruvate and lactate. To evaluate gluconeogenesis, a pyruvate tolerance test (PTT) was performed in WT (n = 9) and *Phkb*^−/−^(n = 6) mice (Figure 2G). *Phkb^−/−^* mice showed a drastic increase of blood glucose through 60 min (71% increase) after the pyruvate injection, after which, the blood glucose began to gradually decrease over the last 60 min. To a lesser extent, WT mice also show an increase in blood glucose levels following the pyruvate injection (63% increase); however, the gradual decrease in blood glucose was seen after 30 min post injection. These results suggest gluconeogenesis may be upregulated in *Phkb^−/−^* mice in a fasted state, while only a partial amount of glycogen could be degraded. Gene expression analysis of key enzymes of gluconeogenesis support these findings. In comparison of hepatic gene expression from non-fasted, 2 h-fasted, and 6 h-fasted liver, fructose 1,6 bisphosphatase (*Fbp1*), a key-enzyme of gluconeogenesis, was significantly upregulated at 6 h of fasting. In addition, Aldolase B (*Aldob*) showed elevation, but it was not significant while *mG6pc* expression was slightly decreased (Figure 3A). Glycolysis (Figure S1A) and glycogenolysis (Figure S1B) related genes were not changed or slightly decreased except moderate increase in *Pygl*^−/−^. Lipogenesis, lipid degradation, and transcriptional regulation related genes were evaluated to investigate fatty acid metabolism. Although there were no significantly changed genes in WT or *Phkb^−/−^* mice, increased volatility was observed in *Phkb^−/−^* mice for fatty acid synthesis, degradation, transcriptional regulation, and transport related genes (Figure S1C,D).

### 2.4. Phkb^−/−^ Exhibit Excessive Glycogen Accumulation with a Mild Profibrogenic Phenotype

**Figure 4:**
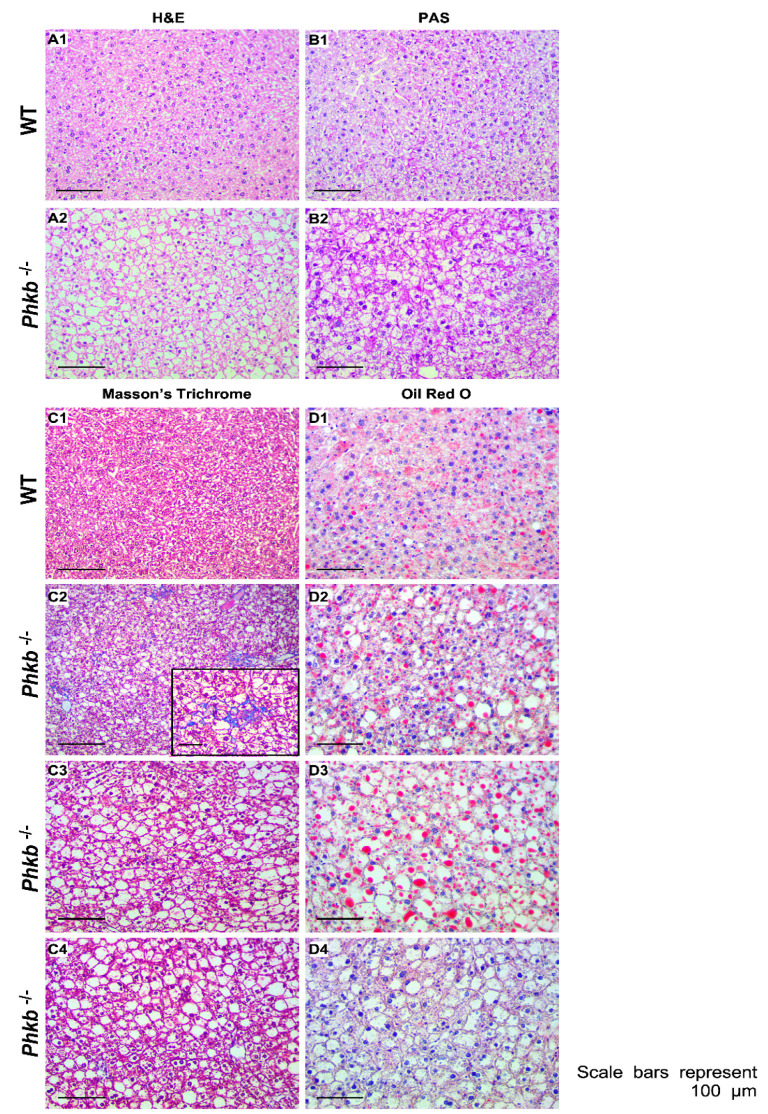
Histologic analysis of livers from WT and *Phkb^−/−^* mice. Representative images of liver sections stained with H&E, PAS, Masson’s trichrome, and Oil Red O in old WT mice (**A1**,**B1**,**C1**,**D1**), old *Phkb^−/−^* mice (**A2**,**B2**,**C2**–**C4**,**D2**–**D4**). With Masson’s Trichrome staining, an individual old *Phkb^−/−^* mouse exhibited minimal collagen deposition in minimal collagen deposition in perisinusoidal areas (**C2**); the images in (**C2**) present higher magnification views of the image of (**C2**). Scale bars represent 100 µm.

Histological analyses by H&E confirmed extensive hepatocellular vacuolar change consistent with glycogen accumulation in *Phkb^−/−^* mice (WT = 6, *Phkb*^−/−^ = 6; Figure 4A1,A2). Liver pathology was assessed based on previous histopathological reports reported in *Pygl*^−/−^ mice [19]. Periodic-acid Schiff (PAS) staining revealed glycogen deposits (WT = 6, *Phkb^−/−^* = 6; Figure 4B,B2). Old age *Phkb^−/−^* mice (5 of 6) showed similar patterns of minimal to mild collagen deposition in perisinusoidal, perisubscapular, and periportal areas compared to age-matched controls in Masson’s trichrome staining (WT = 6; Figure 4C1–C4). Additionally, Oil Red O staining revealed *Phkb^−/−^* mice had similar or lower hepatic triglyceride (WT = 9, *Phkb^−/−^* = 10; Figure 2H) and lipid accumulation (WT = 6, *Phkb^−/−^* = 6; Figure 4D1–D4). In our previous report, *Pygl*^−/−^ mice demonstrated upregulated gene expression of profibrogenic markers which correlated with minimal to regionally severe collage deposition by old age [19]. To determine if *Phkb^−/−^* mice express a similar profibrogenic profile because of impaired glycogenolysis, we measured the relative mRNA levels of profibrogenic markers. Notably, *Phkb^−/−^* mice display increased mRNA levels of *Ctgf*, and Col1a1 at the 2 h fasted and nonfasted time points, respectively (Figure 3B). In addition, investigation on profibrogenic and inflammation related gene showed no significant changes (Figure S1E,F).

## 3. Discussion

The liver has a major role in energy homeostasis of the body. To maintain energy balance, it synthesizes, stores and distributes nutrients such as carbohydrates, lipids, and vitamins. With its other multiple functions, it also plays a critical role in regulating the body’s metabolic homeostasis through glycogenolysis and gluconeogenesis [26]. In glycogenolysis, the phosphorylase kinase (PHK) activates the liver glycogen phosphorylase (PYGL), triggering a conformational change of the inactive to active form of PYGL and promoting glycogenolysis. As a result, glycogenolysis generates the production of free glucose-1-phosphate monomers via cleavage of the α-(1,4) glycosidic linkages in glycogen chains for energy metabolism [5]. This is the rate limiting step of glycogenolysis and a deficiency in PYGL can causes GSD-VI and the *Pygl^−/−^* mice had been previously described [25]. Due to its critical role in glycogenolysis, we postulated that deletion of *PHK* may mimic the GSD-IX phenotype reported in clinical cases [1,14]. As *Phkg2*^−/−^ mice successfully recapitulated human GSD-IX-γ [21], here we characterized the metabolic abnormalities associated with GSD-IX-beta, utilizing a knockout mouse harboring a deficiency in the PHK beta subunit and studied its role in the underlying disease mechanism. Through phenotypical analysis, with hepatomegaly (Figure 1C,D), sub-normal level of fasting glucose profile (Figure 2A), and significantly elevated blood ketone levels (Figure 2B), we confirm that *Phkb^−/−^* mouse perfectly mimics GSD-IX-beta deficiency and it enabled further investigations to find disease mechanism underlying PHK-beta deficiency.

Glycogen accumulation gradually occurs during the fed state; however, the glycogen stores are greatly depleted during fasting as the primary source for endogenous glucose production [27]. To quantify the variations in the mechanism of glycogenolysis effect, we measured hepatic glycogen and glucose through 6 h of fasting. In contrast to *Pygl*^−/−^ mice, *Phkb^−/−^* mice were able to make glucose from the partial degradation of glycogen (Figure 2D,E) which is confirmed by reduced activity of glycogen phosphorylase observed in *Phkb^−/−^* mice liver while minimal or no activity observed in *Pygl*^−/−^ mice (Figure 2C). This capability of endogenous glucose production by partial glycogenolysis in the liver of *Phkb^−/−^* mice can explain the mild phenotypes of GSD-IX-beta [7,10].

The observation of lower concentration of blood glucose and sub-normal level of endogenous glucose production in *Phkb^−/−^* mice led us to investigate alternative metabolic pathways that compensate the energy needs in this mouse model. During prolonged fasting, hepatocytes utilize alternative sources such as pyruvate or lactate for glucose production as gluconeogenesis. The results of Pyruvate Tolerance Test (Figure 2G) and gene expression analysis revealed *Phkb^−/−^* mice have significantly elevated gluconeogenesis pathway while glycogenolysis is limited (Figure 2G and Figure S3A). We also observed mild increase in ALT, AST (Figure 2F), and elevated uric acid in blood (Figure S2B). Amino acids are the major substrates for hepatic gluconeogenesis during fasting. With high concentration of alanine, transamination is the rate limiting step of gluconeogenesis [28]; therefore, the moderate elevation of ALT and uric acid may be the outcome of increased usages of alternative gluconeogenic substrates rather than hepatic damage signal in *Phkb^−/−^* mice. We also found *Phkb*^−/−^ mice maintain low level of hepatic TG level while blood level of TG is slightly elevated and significantly elevated at 6 h of fasting (Figure 2H and Figure S2A). Combined with the lipogenic and lipid degradation related gene expression profile (Figure S2C), we presume that the hepatocytes of *Phkb*^−/−^ mice also use fatty acid and TG as gluconeogenic precursor and/or cellular energy production during fasting. However, it needs further investigation under more controlled environment. In addition, since the kidney has been known as playing a major role in gluconeogenesis, especially during stress condition [29], future investigation into potential glucose production from sources other than glycogen in the *Phkb^−/−^* mouse kidney may help our understanding of the mild ketotic hypoglycemia phenotypes seen with GSD-IX-beta patients.

As a source of circulating energy during fasting, βOHB is transported to metabolically active tissue including the brain and muscle, where it is later converted into acetyl-CoA. Ketone bodies are also reported to affect regulatory functions of metabolism-related genes and enhance *Phkb^−/−^* insulin sensitivity [30]. As significantly elevated ketone levels were observed, we analyzed insulin concentration and sensitivity (Figure S2C,D). While insulin concentration of *Phkb^−/−^* mice is slightly lower than WT, insulin sensitivity has no significant difference compared to WT.

We had previously observed an increased susceptibility to hepatic fibrosis in aged *Pygl^−/−^* mice [25], and *Phkb^−/−^* mouse shares similar phenotype with *Pygl^−/−^* mouse such as hepatomegaly due to increased accumulation of glycogen in liver. Thus, we have followed various aged *Phkb^−/−^* mice over 40 weeks to examine hepatic pathogenesis. In our observation, we found very minimal profibrogenic phenotypes in the group (Figure 4C1–C4) while glycogen accumulation is similar or slightly higher in *Phkb^−/−^* mice (Figure 2D). Gene expression analysis of fibrosis related genes showed increased *Ctgf* and *Col1a1* genes at certain fasting times suggest *Phkb^−/−^* mice have less severe fibrogenic phenotypes parallel with GSD-IX-beta patients [1].

In summary, the metabolic features of *Phkb^−/−^* mice including hepatomegaly, mild ketotic hypoglycemia, excessive glycogen accumulation, and the potential for increased collagen deposition in the liver match clinical reports of GSD-IX-beta mutations in humans [31]. Different from *Pygl*^−/−^ mice, *Phkb^−/−^* mice are capable of producing glucose through partial glycogenolysis with remaining glycogen phosphorylase activity. The upregulation of alternative pathways, including gluconeogenesis with alternative substrates and potential fatty acid oxidation, are supporting energy homeostasis in *Phkb^−/−^* mice.

We have established the first animal model for GSD-IX-beta that recapitulates human GSD-IX-beta. As animal models have been a critical element in human disease [32]. Through our findings of the detailed phenotypical data, this mouse model can serve as a model system to develop GSD-IX-beta targeted therapies and provide valuable insights on hepatic metabolic/genomic regulation under restricted glycogenolysis.

## 4. Materials and Methods

### 4.1. Animal Studies

All animal studies were performed in accordance with guidelines of the Institutional Animal Care and Use Committee of The University of Connecticut Health Center. All mice were maintained in a pathogen-free animal facility at 22–24 °C under the 12:12 light: dark cycle. Standard rodent chow (Envigo, Madison, WI, USA) and water were provided ad libitum. All animals were weaned at PN21 (day 21) and grouped according to gender. Mice previously generated with a *Pygl*-targeted mutation (GSD-VI) were used to compare glycogenolytic phenotypes to *Phkb* deficient mice [25]. Mice with a *Phkb* null allele (*Phkb^−/−^*) generated by the Knockout Mouse Phenotype Program (KOMP) at the Jackson Laboratory (C57BL/6NJ-*Phkb^em1(IMPC)J^*/Mmjax mouse strain; MMRRC stock #42185) were purchased for the study. Heterozygous mating units were used to generate GSD-IX-beta mice. Littermate (*Phkb^+/+^* or *Phkb^+/−^*) animals were used as wild-type (WT) control mice. In this study 4–6 weeks aged mice were used for phenotypic, metabolic, and gene expression analyses. Insulin and pyruvate tolerance test used 10–12 weeks old mice. Mice 40 weeks and older were used for histological analyses. Mice were fasted for 0, 2, 4, 6, and 8 h hours prior to glucose/ketone determination or fasted 0, 2, and 6 h prior to sacrifice for sample collection.

### 4.2. Genotyping

Tail biopsies were lysed in Tail lysis solution (DirectPCR tail lysis; 20 mg/mL Proteinase K) at 55 °C overnight then at 85 °C for 1 h. Genotyping was performed using Accupower PCR premix (Bioneer, Oakland, CA, USA) tubes. Tail DNA was genotyped by PCR using a primer pair IX-beta forward (5′-GCATTAAACAGCATAAATCCAGA-3′) and IX-beta reverse (5′-TCAAATATATCAAATGATCTC CAAAA-3′), to amplify a fragment of 485 bp in wild-type mice, 485 bp and 156 bp in heterozygous mice and 156 bp in *Phkb^−/−^* mice. GSD-VI mice were genotyped as previously described [25]. All primers were designed with Primer3.

### 4.3. Serum Biochemistry

To determine triglyceride, cholesterol, lactate and uric acid levels in blood, serum was collected from each mouse group. Total cholesterol and uric acid were analyzed using kits (TR13421 and TR24321) obtained from Thermo Fisher Scientific (Waltham, MA, USA). Triglycerides were measured with a Serum Triglyceride Determination Kit (TR0100, Sigma-Aldrich, St. Louis, MO, USA) and lactate measured with a colorimetric/fluorometric kit (K607–100, Biovision, Milpitas, CA, USA). Liver transaminase activity (AST/ALT) was analyzed using colorimetric kits (K753-100 and K752-100, Biovision). To determine serum biochemistry and liver transaminase levels, standard curves for each respective kit were prepared and samples were analyzed according to manufacturer’s instructions using the SpectraMax i3x (Molecular Devices, Sunnyvale, CA, USA).

### 4.4. Fasting Glucose and Ketones

Fasting glucose tests and ketone tests were performed during at 0, 2, 4, 6, and 8 h. Blood glucose levels were measured using a blood glucose meter and glucose cuvettes (HemoCue Glucose 201 System; HemoCue, Brea, CA, USA) and blood ketones (β-hydroxybutyrate) concentration measured using blood ketone meter and ketone strips (Precision Xtra; Abbott Laboratories, Abbott Park, IL, USA).

### 4.5. Histopathology of Phkb^−/−^ Mice

Samples of liver from old *Phkb*^−/−^ and WT mice were fixed in 10% neutral buffered formalin and embedded in paraffin using standard methods (Histoserv, Inc., Germantown, MD, USA). Adjacent 4–5 µm sections stained with hematoxylin and eosin (H&E) and Masson’s trichrome were evaluated. Liver sections were also stained by periodic acid Schiff (PAS) for evaluation of glycogen contents.

### 4.6. Liver Glycogen, Free Glucose, and Triglycerides Contents Determination

To measure hepatic glycogen content, 10 mg of liver tissues obtained from nonfasted, 2 h and 6 h fasted WT *Pygl^−/−^* and *Phkb^−/−^* mice were homogenized. Hepatic glycogen was determined from the liver lysate using a Glycogen Colorimetric/Fluorometric assay kit (K646-100, BioVision). Hepatic free glucose from liver homogenates was determined using a D-Glucose assay kit from Megazyme (Chicago, IL, USA). To determine glycogen and hepatic free glucose content, Glycogen and D-Glucose standard curves were prepared, and samples were analyzed using the SpectraMax i3x (Molecular Device). Hepatic triglycerides were measured using a colorimetric Triglyceride Quantification Kit (K622-100, Biovision). 100–200 mg of liver tissue was homogenized in RIPA buffer containing protease inhibitor cocktail. Crude extract was deproteinized according to kit instructions. Deproteinized hepatic lysates were used to measure Triglycerides (TG).

### 4.7. Liver Glycogen Phosphorylase Activity Assay

Hepatic glycogen phosphorylase activity was measured using a colorimetric Glycogen Phosphorylase Assay Kit (ab273271, Abcam, Waltham, MA, USA) that measures the appearance of G1P when excess substrate is present. To measure glycogen phosphorylase activity, liver samples (50 mg) were homogenized in the assay buffer provided in the kit and centrifuged to use soluble materials. The samples were incubated with glycogen to detect the conversion of glycogen into G1P that produces colorimetric byproduct (OD 450 nm). Standard and the samples were analyzed using the SpectraMax i3x (Molecular Device).

### 4.8. Gene Expression Analysis

Total RNAs were isolated from the livers with the use of the TRIzol Reagent (Invitrogen, CA, USA) and RNeasy Protect Mini Kit (74126, QIAGEN, Germantown, MD, USA) 74126) according to the manufacturer’s instructions. Using iScript gDNA Clear cDNA Synthesis Kit (Bio-Rad Laboratories, 172-5035), cDNA was synthesized. The mRNA expression was quantified by CFX96 real-time PCR detection system (Bio-Rad Laboratories, Hercules, CA, USA). Data were analyzed using the CFX Maestro^TM^ software (Bio-Rad Laboratories) and normalized to the mouse ribosomal protein L19 (Rpl19) mRNA expression. The PrimePCR qPCR Assay probes (Bio-Rad Laboratories) used are summarized in Table S1.

### 4.9. Insulin Tolerance Test and Pyruvate Tolerance Test

To measure insulin action and glucose disposal, all mice (10 to 12-week-old *Phkb*^−/−^ and WT) were fasted for 6 h at the start of the light cycle. Glucose concentrations were measured at baseline (time = 0) and monitored every 15 to 30 min for 120 min following intraperitoneal insulin injection (0.5 IU/kg, Sigma Aldrich Cat #I1882) or glucose bolus (2.0 g/kg, Fisher Scientific) using a blood glucose meter and glucose cuvettes (HemoCue Glucose 201 System, HemoCue). To determine the endogenous glucose contribution from hepatic gluconeogenesis, all mice (10 to 12-week-old *Phkb*^−/−^ and WT) were fasted for 16 h overnight. At 16 h. (Time = 0), basal glucose levels were recorded at baseline prior to intraperitoneal administration of a pyruvate bolus (2.0 g/kg, Sodium Pyruvate, Sigma Aldrich). Glucose concentrations were monitored every 30 min for 120 min.

### 4.10. Statistical Analysis

Animal characterization studies, glycogen content and serum biochemistry were analyzed by unpaired *t*-tests using the GraphPad Prism Program, version 7 (GraphPad Software Inc., San Diego, CA, USA). For gene expression studies, the normality of the mRNA expression data was first examined. As some gene expression data deviate from normality, Wilcoxon Rank Sum Tests were performed, using SAS version 9.4 (SAS, Inc., Cary, NC, USA) to validate initial analyses by unpaired t-tests using GraphPad Prism Program, version 7 (GraphPad Software Inc., San Diego, CA, USA). All tests were two-sided and values of *p* < 0.05 were considered statistically significant. For metabolic studies, relative change in glucose levels (to baseline) was plotted using GraphPad Prism Program, version 7 (GraphPad Software Inc., San Diego, CA, USA).

## Data Availability

Not applicable.

## Supplementary Materials

The following supporting information can be downloaded at: https://www.mdpi.com/article/10.3390/ijms23179944/s1.
