# Supplementary material for: A Mouse Model of Glycogen Storage Disease Type IX-Beta: A Role for Phkb in Glycogenolysis
Source: Int J Mol Sci. 2022 Sep 1;23(17):9944. doi: 10.3390/ijms23179944 (PMC9456097; doi:10.3390/ijms23179944)
Supplement: Supplementary file 1 [file ijms-23-09944-s001.zip › ijms-1885608-supplementary.pdf]

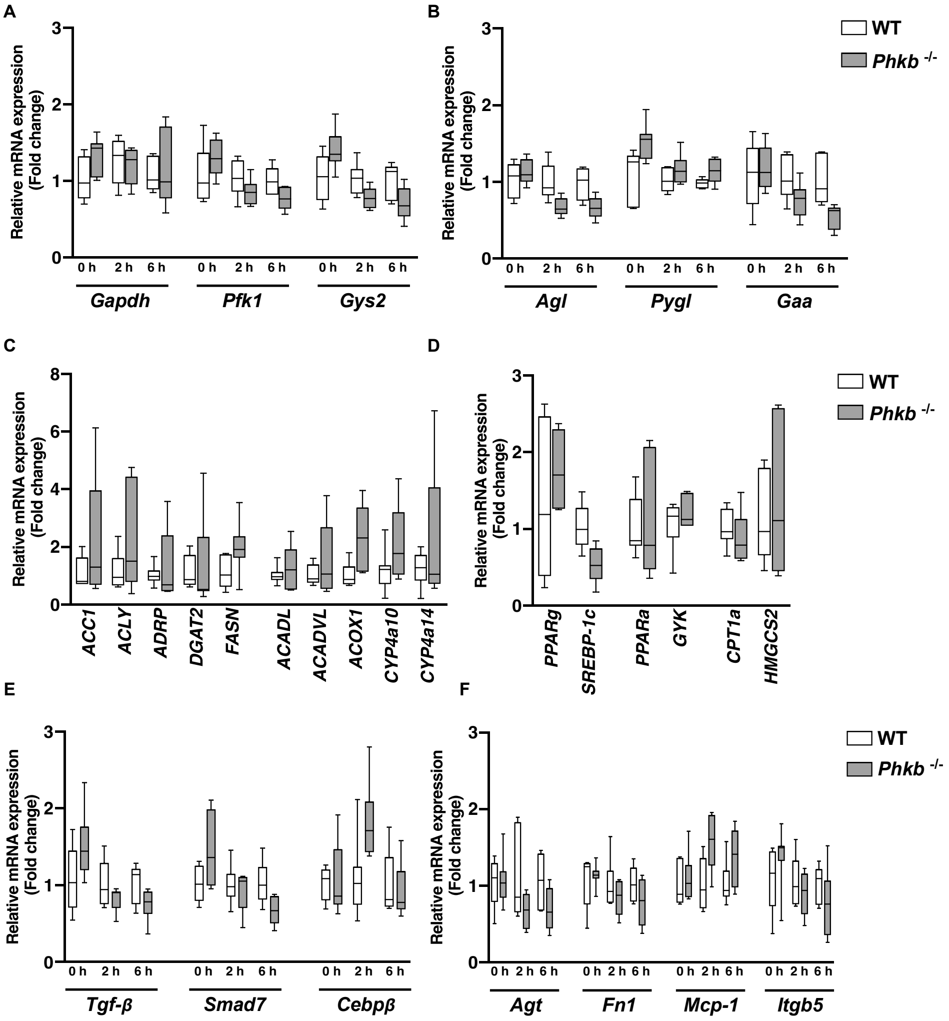

Figure S1. Expression profiles of fibrosis, glycolysis, glycogenolysis, lipogenesis, and lipid degradation related genes in WT and *Phkb*$^{-/-}$ mice. Quantification of hepatic mRNA related to (A) Glycolysis (*Gapdh, Pfk1.* and *Gys2*) and (B) Glycogenolysis (*Agl, Pygl*, and *Gaa*). WT (0 hr, n=5, 2 hr, n=7, and 6 hr, n=5)and *Phkb*$^{-/-}$(0 hr, n=11, 2 hr, n=6, and 6 hr, n=5) mice. (C) Lipogenesis (genes (*Acc1, Acly, Adrp, Dgat2*, and *Fasn*) and lipid degradation related genes (*Acadl, Acadvl, Acox1, Cyp4a10, and Cyp4a14*), (D) Transcriptional regulator and transporters in WT (n=7) and *Phkb*$^{-/-}$(n=7) mice. (E) signaling pathways of fibrosis (*Tgf-β, Smad7,* and *Cebp-β).* (F) profibrotic (*Agt* and *Fn1)* and inflammation related genes (*Mcp-1* and *Itgb5*). WT (0 hr, n=5, 2 hr, n=7, and 6 hr, n=6)and *Phkb*$^{-/-}$(0 hr, n=10, 2 hr, n=6, and 6 hr, n=6) mice. Abbreviations:*TGFb*, Transforming growth factor beta; *Smad7*, Mothers Against Decapentaplegic Homolog 7, *Cebpb*, CCAAT/Enhancer-Binding Protein Beta; *Agt*, Angiotensinogen; *Fn1*, Fibronectin 1; *Il-6*, Interleukin-6; *Mcp-1*, Monocyte chemoattractant protein 1; *Itgb1*, Integrin Subunit Beta 1 *Gapdh*: Glyceraldehyde-3-Phosphate Dehydrogenase; *Pfk1*, Phosphofructokinase, muscle; *Gys*2, Glycogen Synthase 2; *Agl*, Amylo-Alpha-1, 6-Glucosidase, 4-Alpha-Glucanotransferase (Glycogen Debranching Enzyme); *Pygl*, Glycogen Phosphorylase L; *Gaa*, Alpha Glucosidase; *ACC1*, Acetyl-CoA Carboxylase Alpha; *Acly*, ATP Citrate Lyase; *ADRP*, Adipose Differentiation-Related Protein; *DGAT2*, Diacylglycerol O-Acyltransferase 2; *FASN*, Fatty Acid Synthase; *ACADL*, Acyl-CoA Dehydrogenase Long Chain; *ACADVL*, Acyl-CoA Dehydrogenase Very Long Chain; *ACOX1*, Acyl-CoA Oxidase 1; *CYP4a10*, Cytochrome P450 Family 4 Subfamily A Polypeptide 10; *CYP4a14*, Cytochrome P450 Family 4 Subfamily A Polypeptide 14; *PPARg*, Peroxisome Proliferator Activated Receptor Gamma; *SREBP-1*, Sterol Regulatory Element-Binding Protein 1; *PPARa*, Peroxisome Proliferator Activated Receptor Alpha; *GYK*, Glycerol Kinase; *CPT1a*, Carnitine Palmitoyltransferase 1A; *HMGCS2*, 3-Hydroxy-3-Methylglutaryl-CoA Synthase 2

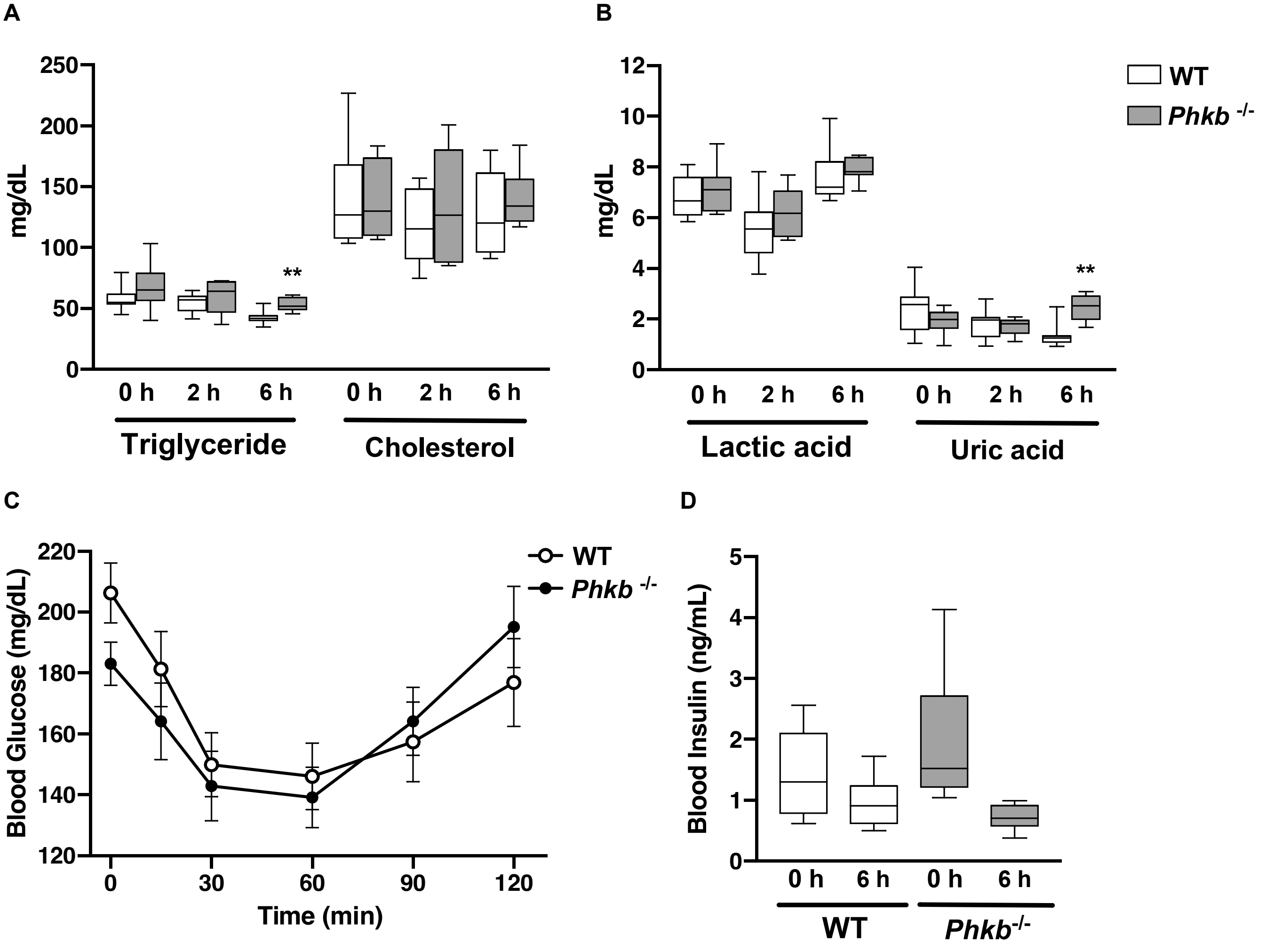

Figure S2. Blood metabolite concentration and insulin. The box-and-whisker plots showing serum **(A)** triglycerides and cholesterol. **(B)** lactic acid and uric acid. WT (0 hr, n=9, 2 hr, n=9, and 6 hr, n=9)and *Phkb*$^{-/-}$(0 hr, n=9, 2 hr, n=6, and 6 hr, n=7) mice for plot (A) and (B). **(C)** Insulin tolerance profiles of WT (n=8) and *Phkb* $^{-/-}$ mice (n=8) treated with 0.5 IU/kg of insulin. **(D)** Non fasted and 6 hours fasted blood insulin levels in WT(0 hr, n=9 and 6 hr, n=9) and *Phkb* $^{-/-}$ (0 hr, n=9 and 6 hr, n=7) mice. ***P* < 0.01.

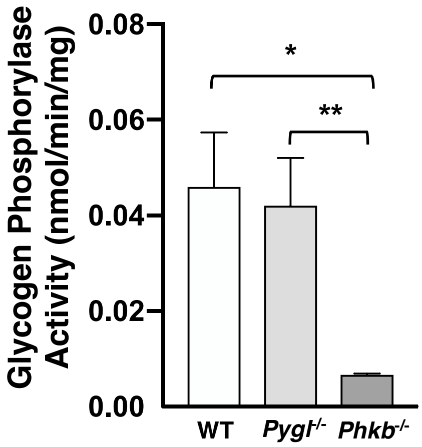

Figure S3. Enzymatic activity of glycogen phosphorylase (PYGL) in wild-type (n=4; WT, white bar), *Pygl*$^{-/-}$ (n=3, light grey bar) and *Phkb* $^{-/-}$ (n=4, grey bar) mice.

Table S1. PrimePCR qPCR Assay probes

Name	Unique Assay ID	Supplier
Mouse ribosomal protein mL19 (Rpl19)	qMmuCEP0042442	Bio-Rad Laboratories
Liver glycogen phosphorylase (Pygl)	qMmuCIP0033749	Bio-Rad Laboratories
Connective tissue growth factor (Ctgf)	qMmuCEP0053713	Bio-Rad Laboratories
Interleukin-6 (IL-6)	qMmuCID0005613	Bio-Rad Laboratories
Chemokine ligand 5 (Ccl5; RANTES)	qMmuCID0021047	Bio-Rad Laboratories
Collagen type 1 alpha 1 (Col1a1)	qMmuCEP0052648	Bio-Rad Laboratories
Phosphorylase Kinase Beta (Phkb)	qMmuCED0049220	Bio-Rad Laboratories
Monocyte chemoattractant protein 1 (Mcp-1)	qMmuCED0048300	Bio-Rad Laboratories
Transforming growth factor b1 (Tgf-β1)	qMmuCID0017320	Bio-Rad Laboratories
Tumor necrosis factor alpha (Tnf-α)	qMmuCED0004141	Bio-Rad Laboratories
Fibronectin 1 (Fn1)	qMmuCID00019534	Bio-Rad Laboratories
Mouse Glucose-6-Phosphatase (mG6pc)	qMmuCID0018783	Bio-Rad Laboratories
Fructose-Bisphosphatase 1 (Fbp1)	qMmuCED0051060	Bio-Rad Laboratories
Aldolase, Fructose-Bisphosphate B (Aldob)	qMmuCED0039716	Bio-Rad Laboratories
Mothers Against Decapentaplegic Homolog 7 (Smad7)	qMmuCED0046593	Bio-Rad Laboratories
CCAAT/Enhancer-Binding Protein Beta (Cebpb)	qMmuCED0050360	Bio-Rad Laboratories
Angiotensinogen (Agt)	qMmuCID0019397	Bio-Rad Laboratories
Integrin Subunit Beta 1 (Itgb5)	qMmuCED0045777	Bio-Rad Laboratories
Glyceraldehyde-3-Phosphate Dehydrogenase (Gapdh)	qMmuCED0027497	Bio-Rad Laboratories
Phosphofructokinase, muscle (Pfk1)	qMmuCID0018472	Bio-Rad Laboratories
Glycogen Synthase 2 (Gys2)	qMmuCED0047720	Bio-Rad Laboratories
Glycogen Debranching Enzyme (Agl)	qMmuCID0005882	Bio-Rad Laboratories
Alpha Glucosidase (Gaa)	qMmuCED0046677	Bio-Rad Laboratories
Acetyl-CoA Carboxylase Alpha (Acc1)	qMmuCID0006041	Bio-Rad Laboratories
ATP Citrate Lyase (Acly)	qMmuCED0044791	Bio-Rad Laboratories
Adipose Differentiation-Related Protein (ADRP)	qMmuCID0016776	Bio-Rad Laboratories
Diacylglycerol O-Acyltransferase 2 (DGAT2)	qMmuCID0012338	Bio-Rad Laboratories
Fatty Acid Synthase (FASN)	qMmuCED0045676	Bio-Rad Laboratories
Acyl-CoA Dehydrogenase Long Chain (ACADL)	qMmuCID0018569	Bio-Rad Laboratories
Acyl-CoA Dehydrogenase Very Long Chain (ACADVL)	qMmuCED0004059	Bio-Rad Laboratories
Acyl-CoA Oxidase 1 (ACOX1)	qMmuCID0016828	Bio-Rad Laboratories
Cytochrome P450 Family 4 Subfamily A Polypeptide 10 (CYP4a10)	qMmuCED0025157	Bio-Rad Laboratories
Cytochrome P450 Family 4 Subfamily A Polypeptide 14 (CYP4a14)	qMmuCID0013174	Bio-Rad Laboratories
Peroxisome Proliferator Activated Receptor Gamma (PPARg)	qMmuCID0018821	Bio-Rad Laboratories
Sterol Regulatory Element-Binding Protein 1 (SREBP-1c)	qMmuCID0009315	Bio-Rad Laboratories

Peroxisome Proliferator Activated Receptor Alpha (PPARa)	qMmuCED0046526	Bio-Rad Laboratories
Glycerol Kinase (GYK)	qMmuCID0040086	Bio-Rad Laboratories
Carnitine Palmitoyltransferase 1A (CPT1a)	qMmuCED0045595	Bio-Rad Laboratories
3-Hydroxy-3-Methylglutaryl-CoA Synthase 2 (HMGCS2)	qMmuCID0016594	Bio-Rad Laboratories
